# Editorial: Gram-Negative Pathogenesis

**DOI:** 10.3389/fmicb.2021.813062

**Published:** 2021-12-13

**Authors:** Hidetada Hirakawa, Christophe Bordi, Haruyoshi Tomita

**Affiliations:** ^1^Department of Bacteriology Gunma University, Graduate School of Medicine, Maebashi, Japan; ^2^Laboratoire d'Ingénierie des Systèmes Macromoléculaires, Aix-Marseille University, CNRS, Marseille, France; ^3^Laboratory of Bacterial Drug Resistance Gunma University, Graduate School of Medicine, Maebashi, Japan

**Keywords:** bacterial pathogenesis, virulence, antimicrobial resistance (AMR), gram-negative, molecular genetics and genomics

Although antimicrobial agents are commonly used for the treatment of bacterial infections, certain Gram-negative bacteria, such as *Pseudomonas aeruginosa*, are innately tolerant to many conventional antimicrobial agents (Poole, 2011). The spread of bacterial strains that have acquired resistance to drugs is a threat in antibacterial chemotherapy. For this reason, we need to establish alternative chemotherapy options that combat bacterial drug resistance/tolerance, such as new classes of antibacterial agents and vaccines.

Gram-negative pathogens produce a variety of virulence proteins required for their pathogenesis and infection. These proteins are considered potential targets for antimicrobial chemotherapy and vaccination. Many scientists are working on examining the roles of these virulence proteins in hosts and how bacterial virulence can be controlled. This Research Topic aimed to provide up-to-date information from basic studies in Gram-negative human pathogens focusing on subjects as characterizing functions of virulence proteins and proposing new idea to treat infections.

The Type III secretion system (T3SS) is a well-studied subset of virulence proteins. This system is composed of a needle-like structural transporter complex and effector proteins secreted via the transporter complex (Galan and Wolf-Watz, 2006). Effector proteins are translocated into the host cells, and induce cell death by disturbing the cell activity. EscV is the largest component of the transporter complex, and highly conserved in *E. coli* pathogenic strains. Mitrović et al. conducted a functional domain analysis within this protein. They identified some amino acid residues, in a transmembrane region, required for oligomerization of this protein and formation of the transporter complex. This finding may provide insights into how the T3SS transporter complex is organized and its activity is disrupted by a target molecule. ExoY is one of the effector proteins produced by *P. aeruginosa*. Its role is poorly characterized compared to other effectors. Silistre et al. showed that the recombinant ExoY has the nucleotidyl cyclase activity. Interestingly, this protein presumably neutralizes the cytotoxicity of *P. aeruginosa*; therefore, it may be a potential repressor molecule to attenuate bacterial virulence.

OmpR was originally characterized as a transcriptional regulator for *omp* genes. It is activated under osmotic stress. Lucchini et al. showed that OmpR is involved in pathogenesis of adherent-invasive *E. coli* (AIEC), a gut pathogen involved in the pathogenesis of Crohn's disease (Darfeuille-Michaud, 2002). The *ompR* mutant of AIEC is highly susceptible to bile salts and colonized in the gut of mice with a low efficiency. The authors suggested that OmpR inhibition may be a good option to prevent exacerbation of Crohn's disease mediated by AIEC infections.

LuxR is known as a group family of transcriptional regulators produced by many Gram-negative bacteria. This family proteins are activated when bacterial cell population density reaches a threshold, then alters the expression of subsets of genes including some genes associated with bacterial pathogenesis (This process is termed “Quorum sensing”) (Fuqua et al., 1994). Zhong et al. found a new LuxR family protein (they named it “RobA”) from *Vibrio parahaemolyticus* and identified genes regulated by RobA in RNA-sequencing analyses. This protein suppresses biofilm formation by reducing expression of genes, including *epsA-J* that encodes a set of enzymes for exopolysaccharides production, via several Quorum sensing-dependent regulatory proteins. RobA may be a modulator of Quorum sensing activity and biofilm. This finding will aid us to understand Quorum sensing and the pathogenesis of *V. parahaemolyticus*.

Use of alternative model animals is a common approach to estimate *in vivo* pathogenesis in a laboratory. Cholera disease is caused by cholera toxin (CT)-producing *Vibrio cholerae* serotype O1 and O139 strains (Harris et al., 2012). Takahashi et al. isolated a strain that carries an orthologous gene encoding CT, but its serotype is neither O1 nor O139. They characterized this strain by using a rabbit ileal loop model and provided evidence that the production of a new CT variant causes this strain to be highly virulent. This finding cautions us about a new serotype of strain that may cause epidemics in the future. Aerobic vaginitis (AV) is described as a form of bacterial vaginosis that is developed when normal vaginal microflora is significantly dominated by aerobic counterparts, such as *E. coli* (Donders et al., 2011). Fan et al. established a model of virginal infection with *E. coli* in pregnant mice and characterized the pathogenesis of AV caused by *E. coli*. The authors identified host molecules affected by infection. The author's study may provide answers to questions such as why pregnancy increases the risk of AV development and how AV can be prevented and treated.

Colistin is used as a “last resort agent” to treat infections caused by multi-drug resistant Gram-negative bacteria (Pogue et al., 2017). However, since the bacteria acquired resistance to colistin is increasing, there is a need to establish alternative strategies to combat the resistance. Feng et al. proposed a chemotherapy idea to treat bacteria that are resistant to colistin The authors found that auranofin, the anti-rheumatic drug, increases the activity of colistin and that the combination use of colistin with auranofin successfully defeated some colistin-resistant Gram-negative strains including *E. coli, P. aeruginosa*., *Klebsiella pneumoniae*, and *Acinetobacter baumannii*.

This Research Topic contains one review article. Nishino et al. described a series of basic studies on drug efflux pumps of Gram-negative bacteria. Drug efflux pumps contribute to both innate and acquired drug resistance. In addition to its original function, some drug efflux pumps in Gram-negative pathogens including *E. coli, P. aeruginosa*, and *Salmonella enterica*, are required for optimal virulence and fitness in hosts. According to the information based on crystal structures, the activity of drug efflux pumps can be inhibited by a pyridopyrimidine derivative compound, named ABI-PP. The inhibition of drug efflux pumps could be a promised strategy for chemotherapy.

Recent advances in experimental techniques, such as the next-generation sequencing and “omics” progressively revealed a large diversity of virulence factors in Gram-negative pathogens. We need to answer questions concerning their mode of action, how expression and function are regulated in host, and how bacterial virulence can be abolished. We believe that this Research Topic provides some new insights into these subjects.

## Author Contributions

HH, CB and HT wrote the manuscript. All authors contributed to the article and approved the submitted version.

## Conflict of Interest

The authors declare that the research was conducted in the absence of any commercial or financial relationships that could be construed as a potential conflict of interest.

## Publisher's Note

All claims expressed in this article are solely those of the authors and do not necessarily represent those of their affiliated organizations, or those of the publisher, the editors and the reviewers. Any product that may be evaluated in this article, or claim that may be made by its manufacturer, is not guaranteed or endorsed by the publisher.
